# The underestimated role of basophils in Ph^+^ chronic myeloid leukaemia

**DOI:** 10.1111/eci.13000

**Published:** 2018-08-06

**Authors:** Peter Valent, Hans‐Peter Horny, Michel Arock

**Affiliations:** ^1^ Division of Hematology & Hemostaseology Department of Internal Medicine I Medical University of Vienna Vienna Austria; ^2^ Ludwig Boltzmann Cluster Oncology Medical University of Vienna Vienna Austria; ^3^ Institute of Pathology Ludwig Maximilian University Munich Germany; ^4^ LBPA CNRS UMR8113 Ecole Normale Supérieure de Paris Saclay Cachan France; ^5^ Laboratory of Hematology Pitié‐Salpêtrière Hospital Paris France

**Keywords:** basophil leukaemia, basophilia, chronic myeloid leukaemia, prognostication, tryptase

## Abstract

Chronic myeloid leukaemia (CML) is a hematopoietic neoplasm defined by the chromosome translocation t(9;22) and the related oncogene, *BCR‐ABL1*. In most patients, leukaemic cells can be kept under control using BCR‐ABL1‐targeting drugs. However, many patients relapse which remains a clinical challenge. In particular, patients with advanced (accelerated or blast phase) CML have a poor prognosis. So far, little is known about molecular and cellular interactions and features that contribute to disease progression and drug resistance in CML. One key prognostic factor at diagnosis is marked basophilia. However, although basophils are well‐known multifunctional effector cells, their impact in CML remains uncertain. In this article, we discuss the potential role of basophils as active contributors to disease evolution and progression in CML. In particular, basophils serve as a unique source of inflammatory, angiogenic and fibrogenic molecules, such as vascular endothelial growth factor or hepatocyte growth factor. In addition, basophils provide vasoactive substances, like histamine as well as the cytokine‐degrading enzyme dipeptidyl‐peptidase IV which may promote stem cell mobilization and the extramedullary spread of stem and progenitor cells. Finally, basophils may produce autocrine growth factors for myeloid cells. Understanding the role of basophils in CML evolution and progression may support the development of more effective treatment concepts.

## 1 INTRODUCTION

Chronic myeloid leukaemia (CML) is a myeloid stem cell neoplasm characterized by uncontrolled accumulation and expansion of myelopoietic stem and progenitor cells and the reciprocal chromosomal translocation t(9;22) that creates the fusion oncogene *BCR‐ABL1*.^1^, ^2^, ^3^ The resulting oncoprotein, BCR‐ABL1, acts as a cytoplasmic driver exhibiting constitutive tyrosine kinase (TK) activity. BCR‐ABL1 triggers several major downstream signalling molecules, including RAS, phosphoinositide 3‐kinase (PI3K) and signal transducer and activator of transcription 5 (STAT5).^4^, ^5^, ^6^ These molecules and the related oncogenic machinery are considered to play a major role in the evolution and pathogenesis of CML. In line with this assumption, BCR‐ABL1‐targeting drugs, like imatinib, have been applied successfully to suppress growth and survival of neoplastic cells in patients with CML.^7^, ^8^

Based on clinical and laboratory parameters, the course of CML can be divided into a chronic phase (CP), an accelerated phase (AP) and a blast phase (BP). The BP of CML is a terminal phase and characterized by blast cell expansion resembling (secondary) acute leukaemia.^9^, ^10^, ^11^ In the CP of CML, BCR‐ABL1 is a major driver of disease evolution, cell survival and proliferation. By contrast, in AP and BP, additional factors and pro‐oncogenic molecules play a critical role in disease progression and drug resistance.^4^, ^5^, ^6^, ^9^, ^10^, ^11^ A key laboratory feature of patients with advanced CML is marked and sometimes even excessive basophilia.^12^, ^13^, ^14^ In addition, a number of previous and more recent data suggest that marked basophilia is a significant prognostic variable in CML at diagnosis.^15^, ^16^, ^17^, ^18^

Several different mechanisms and molecules have been implicated as potential mediators of acceleration and drug resistance in CML, including survival‐related molecules, (autocrine) growth regulators (cytokines), chemokines, cytokine and chemokine receptors and various signal transduction molecules.^4^, ^5^, ^6^, ^10^, ^11^, ^19^, ^20^ Moreover, increased angiogenesis and fibrosis in the bone marrow (BM) and other hematopoietic tissues have been associated with progression in CML.^21^, ^22^, ^23^, ^24^, ^25^, ^26^

As mentioned before, basophils are one of the key prognostic factors in CML. In particular, progressive basophilia is often followed or accompanied by blast cell expansion and disease acceleration in CML. Furthermore, in various scoring systems, marked basophilia represents a most significant, independent prognostic variable in CML.^15^, ^17^, ^18^ However, although the prognostic impact of basophils is well documented, the actual role of basophils in CML remains obscure. In fact, basophils have long been regarded as functionally irrelevant bystander cells that increase in number during disease acceleration. However, more recently, a number of important cell functions of basophils, potentially relevant to disease progression in CML, have been described. These include, among others, the production and release of angiogenic and fibrogenic cytokines, the expression of cytokine‐degrading surface enzymes and the expression and release of vasoactive substances that may facilitate the extramedullary spread and expansion of myeloid cells in various organ systems.

In the present article, we review the potential functions and roles of basophils in CML, with special emphasis on the impact of these cells as active players in disease acceleration and drug resistance. Moreover, we discuss the effects of various targeted drugs on basophils and basophil‐derived mediators.

## 2 BASOPHIL DIFFERENTIATION IN HEALTHY BM AND IN Ph^+^ CML

Basophils are multifunctional hematopoietic cells that are primarily produced in the BM. In fact, basophils are derived from multipotent stem cells and lineage‐restricted hematopoietic progenitors that can be detected in the BM and in the peripheral blood (PB). A number of different types of colony‐forming progenitors (CFU) give rise to basophils under physiologic conditions.^27^, ^28^ The most prevalent bilineage basophil precursor cell detectable in the BM and PB in healthy subjects is CFU‐eo/baso, a cell that develops into basophils and eosinophils but not into other cells independent of the culture condition and cytokine cocktails applied.^27^, ^28^ In patients with CML, an increased production of basophils and of basophil‐committed CFU is a typical finding.^12^, ^27^ In almost all cases, the criteria for hyperbasophilia (HB: >1000 basophils per microlitre blood^14^) are fulfilled. In accelerated phase CML, massive basophilia (>20% basophils) is often found. However, the diagnostic criteria of (secondary) basophilic leukaemia (HB plus ≥40% basophils) are only fulfilled in a small number of these patients.^14^

A number of different growth factors contribute to the development and differentiation of basophils from their multi‐ and unlineage progenitor cells. In the human system, the most potent basophil differentiation factor is interleukin‐3 (IL‐3).^29^, ^30^, ^31^ This growth factor has been described to induce basophil differentiation and maturation in hematopoietic stem and progenitor cells, but also promotes the viability and activation of mature blood basophils.^29^, ^30^, ^31^, ^32^ Other basophil growth regulators include granulocyte/macrophage colony‐stimulating factor (GM‐CSF), IL‐5, transforming growth factor‐beta (TGF‐ß) and thymic stromal lymphopoietin (TSLP).^33^, ^34^, ^35^ In mature basophils, additional factors and molecules, such as complement factors (C3a, C5a) are involved in the regulation of survival, migration, adhesion and activation.^36^ Most of these cytokines are considered to act on CML basophils in the same way as on normal basophils.^27^, ^32^, ^36^

## 3 PROGNOSTIC ROLE OF BASOPHILS IN CML

A number of studies have shown that basophilia is an independent prognostic feature in Ph+ CML and that basophils increase during disease progression.^12^, ^15^, ^16^, ^17^, ^18^ Therefore, basophilia has been included in most prognostic scoring system in CML.^15^, ^17^, ^18^ In addition, basophilia serves as a diagnostic criterion of the accelerated phase (AP) of CML in the World Health Organization (WHO) classification.^37^, ^38^ In these patients, basophils may be quite immature cells. It has also been described that basophils belong to the malignant (Ph+) clone in CML.^39^ The prognostic value of basophilia was first established in patients receiving hydroxyurea or interferon‐alpha^15^, ^17^, ^18^ and has more recently been confirmed for patients receiving imatinib or other BCR‐ABL1 inhibitors.^18^, ^40^, ^41^

## 4 MARKERS OF BASOPHILS AND THEIR APPLICATION IN CML

As mentioned before, basophils may be quite immature cells and sometimes hypogranulated in AP patients and may therefore escape conventional microscopy. Therefore, basophil markers have been developed and have been applied in patients with CML. These include biochemical markers, like histamine or tryptase, as well as cell surface antigens that can be detected by flow cytometry.^42^

**Figure 1:**
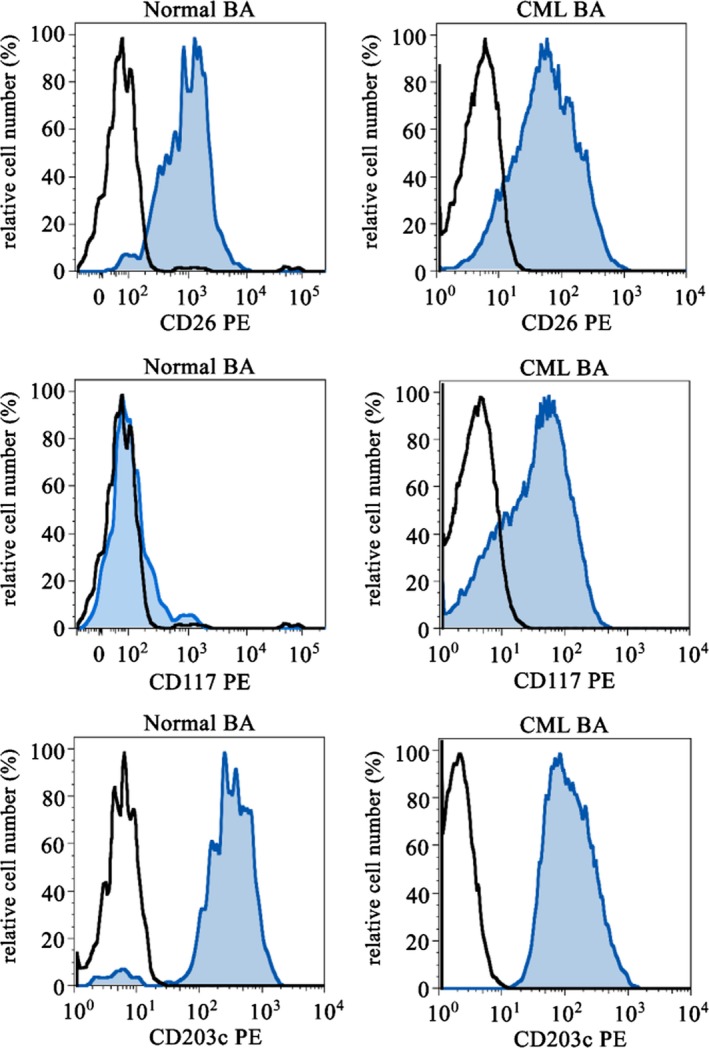
Expression of cell surface antigen on blood basophils. Peripheral blood basophils of healthy normal donors (left images) and of patients with chronic myeloid leukaemia (CML, right images) were stained with antibodies against CD26 (upper panels), CD117 (KIT) (middle panels) and CD203c (lower panels) by multicolour flow cytometry. Basophils were detected by their typical side scatter characteristics, expression of CD123 and CD203c and exclusion of CD34 positivity

The most specific cell surface antigen for basophils is the ectonucleotide pyrophosphatase/phosphodiesterase 3 (ENPP3) also known as CD203c.^43^, ^44^ This antigen is expressed on mature basophils as well as on immature basophil progenitor cells at various stages, including most immature, agranular CD34^+^ basophil progenitor cells.^43^, ^44^ CML basophils also display CD203c (Figure 1). Overall, CD203c is a robust and valuable parameter to quantify the total (immature plus mature) basophil compartment in the PB and BM in healthy individuals and in patients with CML.^14^ However, although basophilia is of major prognostic impact in these patients, CD203c has not yet been tested as a prognostic marker in CML.

Histamine is specifically expressed in basophils among blood leucocytes and is also expressed at all stages of basophil development. Therefore, the total PB leukocyte histamine level, measured in whole blood samples after cell lysis, is a superb biomarker for basophil‐lineage cells in normal controls and in patients with CML.^45^ In fact, in CML patients, total histamine levels are highly upregulated at diagnosis compared to healthy controls and correlate with the presence of basophils.^45^ During successful treatment with imatinib, histamine concentrations in PB cells decrease and return back to normal reference range in those patients who achieve a complete cytogenetic response (CCR).^45^ Furthermore, elevated histamine levels (>100 ng/mL) 3 or 6 months after starting imatinib is associated with lack of optimal response (CCR) and with a reduced probability of survival.^45^

Tryptase is a proteolytic enzyme that is primarily expressed and released in tissue mast cells. However, immature basophils in CML also express and release tryptase.^42^, ^46^ Therefore, serum tryptase levels are elevated in patients with CML when the numbers of immature basophils are high as is typically seen in high‐risk CP and in AP patients.^41^, ^47^ As a result, the serum tryptase level is an excellent biomarker for high‐risk CML.^41^, ^42^ In particular, tryptase levels at diagnosis correlate with basophil counts and are higher in AP or BP patients compared to those with CP CML.^41^, ^42^ Moreover, the rate of progression is higher in patients with elevated tryptase (>15 ng/mL) compared to those with normal tryptase.^41^, ^42^ Finally, when replacing basophils by tryptase levels in the EUTOS score, the prognostication of this score improves significantly.^41^ This is best explained by the fact that very immature hypogranulated basophils (releasing tryptase) are highly prognostic, but are easily missed by conventional microscopy.^41^ Therefore, our recommendation is to include tryptase as improved basophil marker in various prognostic scoring systems.^41^ In addition, tryptase can be measured during follow‐up and serves as a reliable marker of an initial response to BCR‐ABL1‐targeting drugs. In many CML patients, serum tryptase levels decrease below the detection limit during therapy. However, tryptase levels are not recommended as follow‐up marker to quantify minimal residual disease because quantification of BCR‐ABL1 mRNA is a more sensitive approach.

Although a number of most useful basophil markers are available, these parameters are not used in daily practice. This holds true not only for biochemical markers (like tryptase) and flow cytometry markers (CD203c) but also for immunohistochemical parameters. In this regard, it is worth noting that basophils are usually not detectable by conventional cytochemical stains because basophil granules are lost by fixation. However, a number of useful immunohistochemical basophil stains that work in paraffin‐embedded BM section material, have been developed. These markers include the 2D7 antigen and the BB1 antigen, also known as basogranulin.^48^, ^49^ In addition, immature BM basophils express KIT and tryptase.^50^ It has also been described that basophils in CML can be detected and enumerated by 2D7 or BB1 staining and that the numbers of 2D7^+^ and BB1^+^ cells (basophils) correlate with the phase of CML.^48^, ^49^ However, both antibodies may also react with immature eosinophils in the leukaemic BM (P. Valent and H.‐P. Horny, personal observation). Therefore, both markers should be interpreted with caution and additional markers, such as KIT and tryptase (to confirm the basophil lineage) should be applied in patients with CML. Table 1 shows an overview of basophil markers and their potential application in CML.

**Table 1:** Basophil antigens potentially useful as biomarkers in Ph+ CML

Antigen	Application	Role as biomarker in CML
CD203c (ENPP3)	Flow cytometry	Quantification of basophils and their precursor cells in the BM and PB
CD123 (IL‐3RA)	Flow cytometry	Confirms the presence of basophils; also expressed on other leukocytes, including eosinophils, monocytes and myeloid precursor cells
Blood histamine	RIA	Quantification of the total basophil compartment in the PB
Serum tryptase	FIA	Quantification of immature CML basophils at diagnosis; useful for prognostication as individual serum parameter or in the context of CML scores (EUTOS)
Basogranulin (BB1)	IHC	Quantification of basophils in CML on BM section material; may also be expressed in immature eosinophils and neoplastic mast cells
2D7	IHC	Quantification of basophils in CML on BM section material, may also be expressed in immature eosinophils and neoplastic mast cells
BM tryptase	IHC	Confirms the presence of basophils in CML—but is also expressed in normal and neoplastic mast cells
BM KIT (CD117)	IHC	May be expressed on immature CML basophils—but is expressed also on mast cells and stem cells

BM, bone marrow; ENPP3, ectonucleotide pyrophosphatase/phosphodiesterase 3; FIA, fluoro‐immuno‐enzyme assay; IHC, immunohistochemistry; IL‐3RA, interleukin‐3 receptor alpha chain; Ph+ CML, Ph chromosome‐positive chronic myeloid leukaemia; RIA, radioimmuno‐assay.

## 5 BASOPHILS AS UNIQUE SOURCE OF MICROENVIRONMENT‐REMODELLING SUBSTANCES

**Figure 2:**
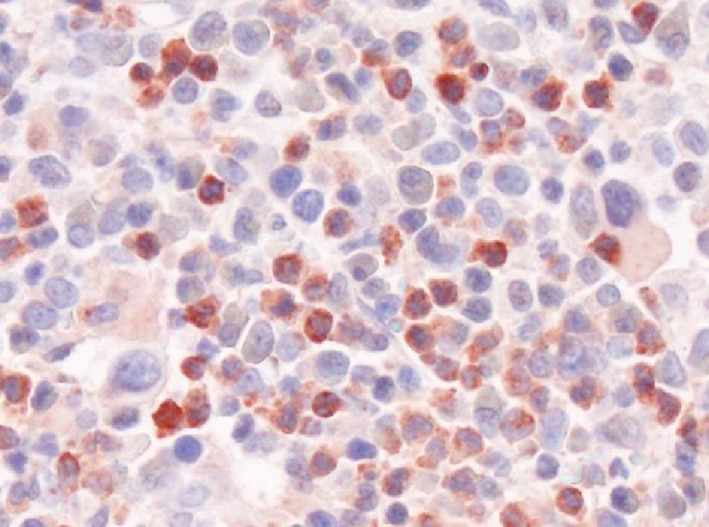
Immunohistochemical detection of HGF in CML basophils. A bone marrow (BM) section of a patient with chronic myeloid leukaemia (CML) in accelerated phase with massive basophilia was stained with an antibody against hepatocyte growth factor (HGF) by indirect immunohistochemistry. Almost all reactive BM cells are basophils. Original magnification: ×600

A number of angiogenic cytokines have been identified in CML cells, including vascular endothelial growth factor (VEGF), basic fibroblast growth factor (bFGF), angiopoietin‐1 (Ang‐1) and matrix metalloproteinases (MMP).^21^, ^22^, ^23^, ^51^, ^52^, ^53^, ^54^ In addition, hepatocyte growth factor (HGF) is expressed in CML cells (Figure 2).^55^, ^56^, ^57^, ^58^ In particular, it has been described that patients with CML exhibit elevated HGF levels in their PB and BM and that expression of HGF in the BM correlates with the microvessel density in BM sections.^55^, ^56^, ^57^, ^58^ Moreover, increased PB levels of HGF correlate with the prognosis in CML.^57^, ^58^ Other studies have shown that HGF is specifically synthesized by CML basophils and that basophil‐derived HGF promotes migration and growth of endothelial cells through a specific receptor.^58^ However, basophils are also known to produce and secrete other angiogenic and fibrogenic cytokines, including VEGF‐A, VEGF‐B and Ang‐1 (Table 2).^59^, ^60^, ^61^, ^62^ Moreover, immature CML basophils produce and release tryptase, a potent mitogen for fibroblasts and endothelial cells.^63^, ^64^, ^65^ Finally, histamine is known to regulate multiple endothelial cell functions, including angiogenesis.^66^, ^67^ All these observations point to a hitherto unrecognized, active, role of basophils (and their products) in the evolution and progression (acceleration) of CML (Table 2). In addition, these data suggest that basophils and their products may serve as potential new therapeutic targets in CML.

**Table 2:** Basophil‐derived mediators and cytokines and their possible role in the pathogenesis of CML

Mediator/antigen	Biological effects	Potential role in the pathogenesis of CML
Tryptase	Fibroblast proliferation Endothelial cell growth	BM fibrosis Increased BM angiogenesis
HGF	Fibroblast proliferation Endothelial cell growth	BM fibrosis Increased BM angiogenesis
Angiopoietin‐1	Endothelial cell growth	Increased BM angiogenesis
VEGF^a^	Endothelial cell growth Vascular permeability‐mediated redistribution of leukocytes	Increased BM angiogenesis Extramedullary spread of leukocytes and stem cells
Histamine	leukocyte homing by selectin‐regulation	Extramedullary spread of leukocytes and stem cells
CD26	Mobilization of myeloid stem and progenitor cells through degradation and inactivation of SDF‐1	Extramedullary spread of stem cells, with consecutive myelopoiesis in various extramedullary organs

BM, bone marrow; CML, chronic myeloid leukaemia; HGF, hepatocyte growth factor; SDF‐1, stroma cell‐derived factor‐1; VEGF, vascular endothelial growth factor.

a Activated basophils reportedly express and release VEGF‐A and VEGF‐B.

So far, little is known about the regulation of synthesis and expression of angiogenic and fibrogenic cytokines in CML cells, including basophils. A number of studies have shown that BCR‐ABL1 is itself involved in the production of VEGF in CML cells.^51^, ^53^, ^54^ In addition, BCR‐ABL1 has been implicated in the production of histidine decarboxylase (HDC) and thus in the synthesis of histamine in CML cells.^68^ However, not all angiogenic and fibrogenic cytokines are produced in CML cells in a BCR‐ABL1‐dependent manner. For example, HGF is produced in CML basophils independent of BCR‐ABL1.^58^ In fact, the biochemical basis underlying expression and release of HGF in basophils in CML remains at present unknown.

## 6 POSSIBLE ROLE OF BASOPHILS IN MODULATING STEM CELL‐NICHE INTERACTIONS

**Figure 3:**
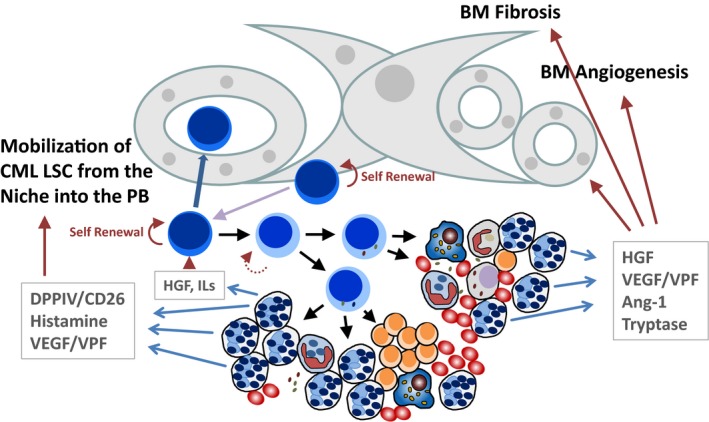
Potential roles of basophils in the bone marrow (BM) of patients with CML. Chronic myeloid leukaemia (CML) is characterized by an increase and mobilization of clonal stem cells, extramedullary myelopoiesis and clonal basophilia. In addition, BM fibrosis and increased angiogenesis are typically found in CML. Basophils are known to produce and secrete several key mediators contributing to the pathogenesis and evolution of CML. These cells also increase in number during disease acceleration. Basophil‐derived dipeptidyl‐peptidase IV (DPPIV = CD26) cleaves stroma cell‐derived factor‐1 (SDF‐1) and thereby facilitates the mobilization of leukaemic stem cells (LSC) out of the niche. Basophil‐derived histamine and vascular endothelial growth factor (VEGF), also known as vascular permeability factor (VPF), can promote the transmigration of CML LSC and may facilitate the redistribution of these cells into the peripheral blood (PB) and may thereby trigger extramedullary myelopoiesis. Basophil‐derived hepatocyte growth factor (HGF) and basophil‐derived interleukins (ILs) may be involved in the regulation of growth and differentiation of CML LSC. Finally, basophil‐derived HGF, VEGF, angiopoietin‐1 (Ang‐1) and tryptase can induce the growth and accumulation of fibroblasts and endothelial cells and thereby can promote angiogenesis and fibrosis in the BM in CML

Leukaemic stem cells (LSC) in CML are characterized by their self‐renewal ability and their capacity to propagate the CML for unlimited time periods.^69^, ^70^, ^71^, ^72^ Contrasting normal stem cells, CML LSC are less capable of homing into BM niches, presumably because of altered interactions with chemotactic cytokines, such as stroma cell‐derived factor‐1 (SDF‐1).^73^, ^74^, ^75^ As a result, CML LSC are considered to redistribute into the blood at high rates, which results in a persistent, marked, extramedullary spread of myelopoietic stem and progenitor cells. One critical molecule regarding LSC redistribution may be CD26, a surface enzyme (dipeptidyl‐deptidase IV = DPPIV) known to cleave SDF‐1 into inactive fragments. In CML, LSC themselves reportedly display CD26.^72^ Most other cell types in the normal BM and CML BM lack CD26. However, normal and CML basophils also display CD26 (Figure 1). The notion that basophils express substantial amounts of CD26 on their surface suggests that these cells may also be involved in SDF‐1 degradation and in the related migratory defect of CML LSC against this cytokine. Indeed, normal and CML stem cells express CXCR4, the receptor for SDF‐1; and disruption of SDF‐1 activity is considered to lead to stem cell mobilization.^71^, ^72^, ^76^, ^77^, ^78^ In this regard, it is worth noting that in patients with severe allergies where basophils may also increase, the numbers of circulating colony‐forming progenitor cells also increase.^79^ There may be also other mechanisms through which basophils can modulate stem cell‐niche interaction. First, as mentioned, basophils are a rich source of vascular growth factors and may thus be able to contribute to stem cell‐niche expansion and increased angiogenesis. Moreover, basophils display many vascular permeability‐augmenting substances, including VEGF (identical with vascular permeability factor, VPF), HGF and histamine.^58^, ^59^, ^60^, ^61^, ^62^ These molecules may well facilitate stem cell redistribution from the BM into the circulation and also from the PB into extramedullary organs (Figure 3). It is also worth noting that basophil‐derived histamine augments selectin expression on endothelial cells which may also contribute to transmigration and homing of myeloid stem and progenitor cells. Finally, basophils may produce and release autocrine growth regulators that act on neoplastic stem and progenitor cells in the CML clone (Figure 3). For example, it has been described that CML stem cells display c‐MET, the receptor for HGF and that basophil‐derived HGF acts as an autocrine growth regulator on CML LSC in the malignant clone.^58^ Indeed, HGF is a well‐known regulator of early myeloid progenitor cells.^80^, ^81^, ^82^

## 7 IMPACT OF BASOPHILS IN PH‐NEGATIVE MYELOPROLIFERATIVE NEOPLASMS (MPN)

Basophils may also increase in number in Ph‐negative MPN, especially in patients with primary myelofibrosis (PMF).^49^, ^83^ In most of these patients, basophilia is mild contrasting the excessive basophilia seen in CML. However, in some patients with PMF, marked basophilia may develop, and in a few cases, secondary basophilic leukaemia has been reported.^84^ These patients have a grave prognosis. Moreover, it has recently been described that absolute basophilia is an adverse prognostic variable in patients with PMF.^85^

## 8 CONCLUDING REMARKS AND FUTURE PERSPECTIVES

Marked basophilia is a pathognomonic feature and a strong prognostic factor in *BCR‐ABL1* ^+^ CML. So far, basophils have been regarded as prognostic bystander cells but not as active players in disease progression. More recently, however, CML basophils and their products have been implicated as active disease‐triggering components of the malignant clone. In fact, basophils produce and secrete a number of relevant disease‐triggering angiogenic, fibrogenic, immunomodulating and stem cell‐active cytokines. In addition, basophils express and release several vasoactive amines, peptides and cytokines involved in the regulation of redistribution, homing and invasion of CML stem and progenitor cells into various extramedullary organs. The process of stem cell mobilization may be further facilitated by basophil‐derived CD26 (DPPIV), a surface enzyme degrading the stem cell homing receptor SDF‐1. Based on these assumptions, the prognostic impact of basophils is confirmed and these cells may now be regarded as more active players and disease‐modifying elements in CML. Application of more specific basophil‐related antigens, such as CD203c, tryptase, BB1 or 2CD7, may assist in accurate basophil quantification at diagnosis and during follow‐up.

## CONFLICT OF INTEREST

The authors declare that they have no conflict of interest in this study.
